# Influence of Additive Firing on the Surface Characteristics, *Streptococcus mutans* Viability and Optical Properties of Zirconia

**DOI:** 10.3390/ma14051286

**Published:** 2021-03-08

**Authors:** Wonjoon Moon, Joo Hyang Park, Han-Ah Lee, Bum-Soon Lim, Shin Hye Chung

**Affiliations:** Department of Dental Biomaterials Science, Dental Research Institute, School of Dentistry, Seoul National University, Seoul 03080, Korea; w.moon@snu.ac.kr (W.M.); coolbox0@snu.ac.kr (J.H.P.); han6936@snu.ac.kr (H.-A.L.)

**Keywords:** zirconia, firing, roughness, contact angle, *S. mutans*, translucency

## Abstract

The purpose of this study was to observe whether the repetitive firing of dental zirconia caused changes in surface characteristics, *S. mutans* viability, and optical properties of zirconia. Dental zirconia blocks were sintered and randomly distributed into seven experimental groups: F0–F6. Except for F0, which only went through sintering, the additive firing was performed in order for F1–F6. Surface roughness, contact angle, *S. mutans* viability by fluorescence, and translucency parameter were measured. They were all highest after sintering (F0) and decreased after additive firings (F1–F6). The additive firing of zirconia after sintering decreased surface roughness, contact angle, *S. mutans* viability, and translucency. The number of firings after the first firing was not found to be critical in surface characteristics, *S. mutans* viability, and optical property. Changes in surface characteristics might have led to a decrease in *S. mutans* viability, while the change of translucency was not clinically significant. This implies that additive firing may prevent secondary caries under zirconia restorations, not compromising esthetic appearance.

## 1. Introduction

Dental zirconia has been widely used for esthetic prostheses that required high physical properties due to its superior mechanical properties and chemical stability compared to conventional ceramic materials [1,2,3]. It has been used not only as all-ceramic restorations, core materials, and orthodontic brackets, but also as an implant material because of its excellent biocompatibility and bone fusion with the alveolar bone [4].

The method of using zirconia for tooth restoration can be divided into two categories. One is its use as a core material; ceramic material is then fabricated on the top of zirconia in a bilayer form (porcelain fused zirconia; PFZ). Due to the optical impermeability of zirconia, it is covered with veneering porcelain. Feldspar is mainly used to reproduce the esthetic characteristics of natural teeth. The veneering process of placing ceramics on the zirconia surface generally requires five stages of firings (750–900 °C) Another method is a pre-colored dental zirconia block that overcomes the porcelain chipping, which is the biggest drawback of PFZ. Zirconia is manufactured and used in a crown shape without ceramic material (full-contour zirconia; FCZ). However, the colored zirconia blocks are also insufficient to reproduce the colors of various teeth, so additional coloring or firing in the dental laboratory is required. Therefore, additive firing is one of the essential parts in the fabrication of dental zirconia prostheses.

Firing process and its effect on properties of zirconia are not fully understood, but there have been several reports on the negative effects of firing. Although the additive firing temperature was lower than the sintering temperature of the core material, it decreased the flexural strength and microhardness of zirconia [5]. In addition, even at lower temperatures, low-temperature degradation was found to be accelerated in yttria-stabilized zirconia implants used in orthopedics [6]. In contrast, one of the clinical advantages of firing was that it improved marginal fit [7]. With regard to translucency, previous studies reported its decrease after repeated firing [8,9], but the clinical significance of such decrease was not fully investigated.

Surface characteristics such as surface roughness and contact angle are closely related [10,11]. Their relationship that an increase in one increases another and vice versa has been well known and shown in the researches regarding dental zirconia [12,13]. To understand the effect of surface roughness and contact angle on zirconia, various processes such as laser scanning, sandblasting, polishing, machining, and heat treatment were performed to produce diverse topography [14,15,16]. However, the effect of repeated firing on the surface roughness and contact angle of zirconia has not been studied yet.

Analysis of surface roughness and contact angle in terms of biofilm accumulation is also important since they affect microbial adhesion to induce biofilm formation [17,18,19]. In particular, increased surface roughness [20,21] and specific surface topography [22] were reported to be major contributing factors of microbial adhesion. Biofilm on dental restorations has been known to have harmful effects since it causes secondary caries and peri-implantitis [23,24,25]. Among various microorganisms participating in biofilm formation and caries, *S. mutans* plays a main role [26]. For successful dental restorations, reducing *S. mutans* adhesion might be important, so there has been research on adhesion of *S. mutans* on zirconia [27,28]. To provide a more favorable surface to reduce *S. mutans* adhesion, reduction in surface roughness and hydrophobicity has been found to be successful [29,30]. However, the previous studies were limited to zirconia without firing, and there have been a lack of studies in relation to adhesion after firing.

Despite the importance and advantage of firing in zirconia, its effect has not been clearly identified in relation to surface roughness, contact angle, *S. mutans* viability, and translucency. In addition, those characteristics have not been integrated to suggest clinical relevance. Therefore, the purpose of this study was to observe whether the repetitive firing of dental zirconia caused changes in surface characteristics, *S. mutans* viability, and optical property of zirconia and to discuss the importance of them in clinical perspective.

## 2. Materials and Methods

### 2.1. Preparation of Specimen

**Figure 1:**
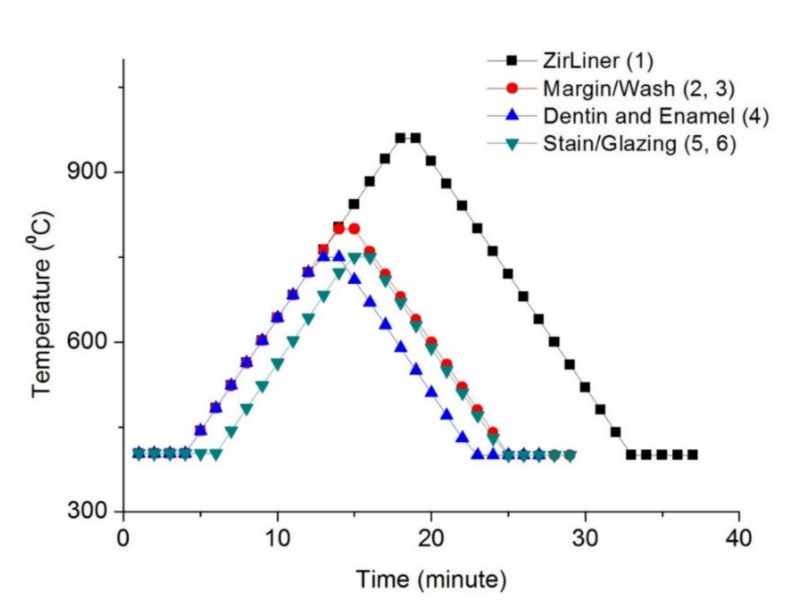
Firing schedule of sintered zirconia (ZirLiner: zirconia lining material).

Dental zirconia blocks (Lava Plus, 3M ESPE, St. Paul, MN, USA) were cut and finished by a low-speed diamond disc (MD-Piano, Struers, Ballerup, Denmark). They were then sintered, up to 1450 °C, according to the manufacturer’s instructions. The specimens were randomly distributed into seven experimental groups: F0 (control; sintering only), F1 (first additive firing (ZirLiner; zirconia lining material)), F2 (second additive firing (Margin)), F3 (third additive firing (Wash)), F4 (fourth additive firing (Dentin and Enamel)), F5 (fifth additive firing (Stain)), and F6 (sixth additive firing (Glazing)); n = 7 each. Except for F0, which only went through sintering, the additive firing was performed for F1–F6 according to the manufacturer’s instructions (Figure 1). They were embedded in an epoxy resin (Cold Mounting Systems Epoxy Systems, Metallurgical Supplies, Buffalo, NY, USA) and went through final finishing and polishing up to 0.06 μm abrasive (LaboPol-5, Struers, Ballerup, Denmark).

### 2.2. Surface Characteristics

For all the groups (F0–F6), surface roughness was measured by confocal laser scanning microscopy (CLSM) (LMS 5-Pascal, Carl Zeiss, Oberhausen, Germany) at 20 × objective to obtain the image field of 500 × 500 μm. High pass filter cut-off ($\lambda_{c}$) of 800 μm and low pass filter cut-off ($\lambda_{s}$) of 2.5 μm were used to determine roughness from noise and waviness. Ra (arithmetic mean roughness of profile), Sa (mean height of the surface), Sz (maximum height of surface), and Sv (maximum pit height of surface) values were obtained for the roughness parameters. Representative surface profiles were also obtained and also contact angles were measured (Phoenix 150, SEO, Suwon, Korea). At room temperature, one drop of distilled water was placed at the center of the zirconia surface and the camera acquired the profile image. A computer software determined the contact angle between the surface and the tangent of the water drop on the zirconia. Roughness and contact angles were measured with three specimens from each group.

### 2.3. Streptococcus Mutans Viability

*Streptococcus mutans* (UA159) was cultured in Brain Heart Infusion Broth (Sigma-Aldrich, St. Louis, MO, USA) at 37 °C of 100% relative humidity. Zirconia specimens were prepared by ultrasonication with ethanol and distilled water. Then *S. mutans* (OD_600_ = 0.5) was cultured over the prepared specimen for 24 h. *S. mutans* was treated by bacterial viability kit (LIVE/DEAD^®^ BacLight^TM^, Invitrogen by Thermo Fisher Scientific, San Jose, CA, USA) for 15 min to be observed under CLSM (LSM700, Carl Zeiss Meditec, Jena, Germany). Based on the images, the area fraction of the fluorescent cells was calculated with computer software (ImageJ, NIH, Bethesda, MD, USA).

### 2.4. Calculation of Translucency

The translucency was measured using the translucency parameter (TP) [31] with three specimens of each group. The CIE L^*^a^*^b^*^ values (L^*^ referred to brightness, a^*^ to redness-greenness, and b^*^ to yellowness–blueness) were measured at the center of each specimen over a black (B) and white (W) background using a spectrophotometer (Ci7600, X-rite, Grand Rapids, IL, USA). Then the TP values were calculated by the following equation:

(1) $$TP={\left[{\left(L^{*}{}_{W}-L^{*}{}_{B}\right)}^{2}+{\left(a^{*}{}_{W}-a^{*}{}_{B}\right)}^{2}+{\left(b^{*}{}_{W}-b^{*}{}_{B}\right)}^{2}\right]}^{1/2}$$

### 2.5. Statistical Analysis

Surface roughness, contact angle, and translucency parameters were analyzed by a nonparametric Kruskal–Wallis test and Mann–Whitney test with Bonferroni correction as they failed the normality test. All statistical analyses were performed with a significance level of 0.05 using SPSS statistics for Windows (version 26.0, IBM, Armonk, NY, USA).

## 3. Results

### 3.1. Surface Characteristics

**Figure 2:**
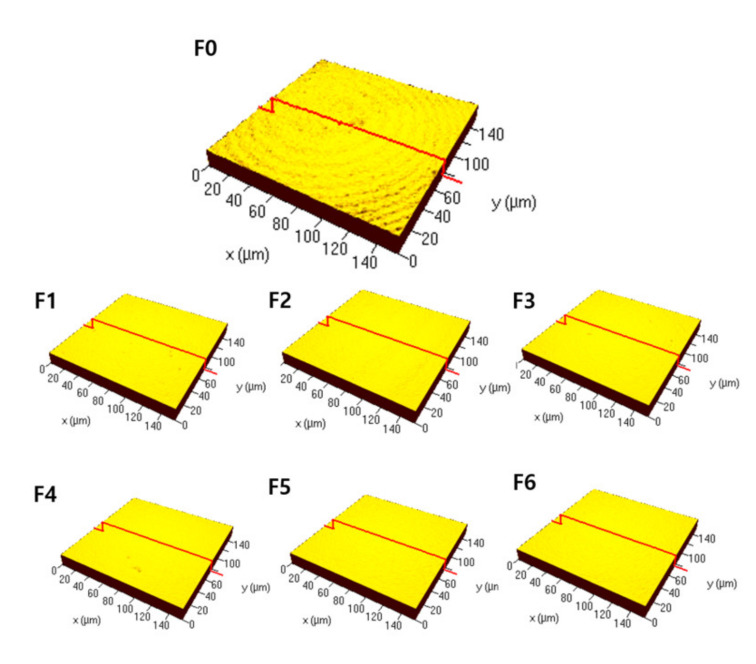
Representative surface profiles with enlarged pictures for the experimental groups (F0–F6); red lines indicate surface profile.

The surface roughness measured as mean height (Sa) was the highest (0.17 (0.15, 0.18) μm) immediately after sintering (F0; control), and the roughness decreased to 0.07 (0.06, 0.07) μm after the first firing (F1). Second firing (F2) (0.06 (0.06, 0.06) μm), third firing (F3) (0.06 (0.06, 0.06) μm), fourth firing (F4) (0.06 (0.06, 0.07) μm), fifth firing (F5) (0.06 (0.06, 0.06) μm), and sixth firing (F6) (0.06 (0.06, 0.07) μm) also decreased the surface roughness compared to F0. For the arithmetic mean roughness of profile (Ra), maximum height of surface (Sz), and maximum pit height of surface (Sv), the same trend as in mean height (Sa) was observed (Table 1). Moreover, representative surface profiles were obtained (Figure 2). It was consistent with the roughness values in that F1–F6, in contrast to F0, had decreased roughness and showed no identifiable surface structures.

**Figure 3:**
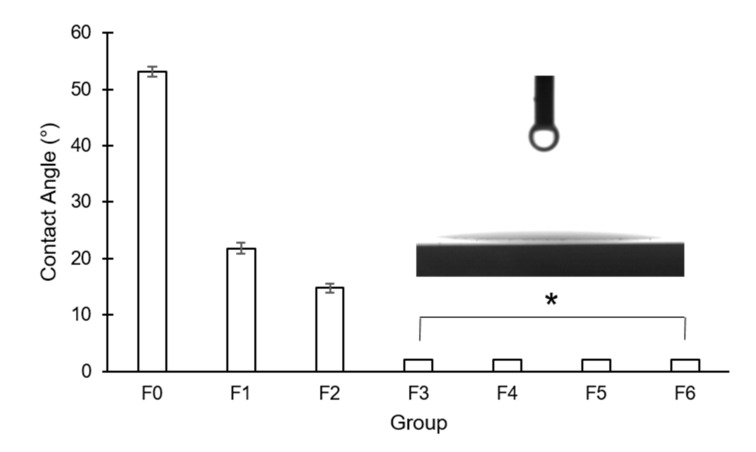
Measurement of contact angles of the experimental groups (F0–F6) (*: The values were less than 10°, indicating their hydrophilic properties).

The contact angle was 53.13 ± 0.82° in F0, and it decreased to 21.83 ± 0.97° in F1 and to 14.75 ± 0.84° in F2. In F3–F6, the contact angle was less than 10°, indicating their hydrophilic properties (Figure 3). There was a significant difference between every pair of the groups (*p* < 0.05).

### 3.2. Streptococcus Mutans Viability

**Figure 4:**
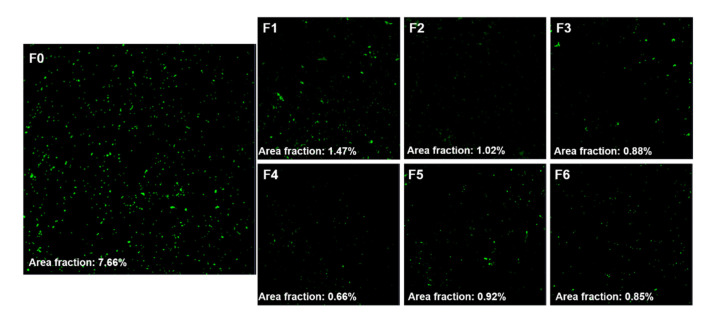
*S. mutans* cultured over zirconia specimens (F0–F6) for 24 h; fluorescence images and the area fraction of fluorescence in F0–F6.

The area fraction of fluorescence, which represented the viability of *S. mutans*, observed with CLSM was the highest in F0 (7.66%), and it decreased to 1.47% (F1), 1.02% (F2), 0.88% (F3), 0.66% (F4), 0.92% (F5), and 0.85% (F6). Its decrease was visualized as a series of fluorescence images (Figure 4).

### 3.3. Optical Property

Median translucency parameters decreased after firing (F1–F6) compared to F0. It was 5.05 in F0 and decreased to 4.39 (F1), 4.21 (F2), 4.43 (F3), 4.66 (F4), 4.53 (F5), and 4.79 (F6) (Table 2).

## 4. Discussion

Dental zirconia undergoes additive firing during the veneering process for esthetic purposes, and their physical properties may change during the process. It was reported that the veneering process reduced mechanical properties such as flexural strength, microhardness [5], and bond strength of ceramic [32]. In this study, surface characteristics, microbial viability, and translucency were newly tested, and firing conditions were more specifically divided (F0–F6). The surface roughness was the highest in F0 and decreased rapidly after the first firing (F1). After that, the roughness did not show radical change according to the number of firing times from the second to sixth firing. To the best of the authors’ knowledge, there have been no previous reports on the decrease of roughness after firing. The possible explanation for the phenomenon was that the microstructure or debris on the surface could have been destroyed and removed due to high energy transmitted by firing processes which were conducted at a maximum of 750–900 °C. This might also explain that there was only a small change in surface topography after the first firing. Decreased roughness in dental zirconia has been known to be more favorable to initial fibroblast adhesion [33]. Fibroblast adhesion has been typically used for testing cytotoxicity of dental materials [34]. Considering its high possibility of contact with surrounding soft tissue, dental zirconia’s lowered roughness might provide higher biocompatibility. 

For the bacteria, on the other hand, increased roughness induces their adhesion due to irregularities and increased surface area [35]. This phenomenon was well presented in various biomaterials [36,37]. In this study, higher roughness in F0 led to higher *S. mutans* viability, so it was consistent with the previous studies. The significance of this study was that such phenomenon was reproduced by repeated firings. Increased roughness and the following hydrophobicity are also well-known [38]. Typically, roughness-based hydrophilicity was obtained after polishing by reducing roughness [39], but the firing, which was a part of zirconia veneering, showed that it could reduce roughness to induce hydrophilicity more efficiently.

To analyze the surface roughness both on a profile and surface, profile roughness parameter (Ra) and areal roughness parameters (Sa, Sz, and Sv) were taken. Although Ra could provide the arithmetic mean of the roughness on a profile, it only measures 2D roughness. To include 3D information about the roughness, the areal parameters were additionally used in the study [40]. First, Sa was measured to give an overall picture of the surface. As a result, the specimens in the study were found to be full of peaks and valleys, so the parameters which were sensitive to peaks and valleys were measured to capture them. Sz values which represented the sum of maximum peak heights and maximum pit heights and Sv values which represented the maximum pit heights, were used to supplement Sa values. If additional feature parameters such as Spd, Spc, etc. can be provided in future studies, it would suggest more information about the surfaces.

The contact angle was significantly reduced after the first and second firing (F1 and F2), and it was consistently smaller than 10° in the following firing. This might be explained in relation to the physicochemistry alteration of the surface. Although there have been no explicit results about the effect of firing, numerous approaches, including oxygen plasma and ultraviolet treatment, were known to induce hydrophilicity by removal of hydrocarbons and insertion of polar groups on the surface [41]. As the firing also conveys heat energy, hydrophilicity might have been obtained by similar mechanisms. Since hydrophilicity of dental zirconia as restorative material may improve its surface adaptation of adhesives, primers, or resin cement [42], firing-induced hydrophilicity may have advantages in the bonding process. However, to utilize the increased hydrophilicity after firing, further studies should be performed to test whether such property is maintained for the intended period of time.

Among the various types of bacteria residing in the oral cavity, *S. mutans* forms a biofilm on the tooth surface and demineralizes it, causing dental caries [43]. Reducing *S. mutans* viability to inhibit biofilm formation is important in preventing dental caries. After firing zirconia in this study, the viability of *S. mutans* was reduced in all stages (F1–F6), which can be said to be a clinical advantage. However, although *S. mutans* is one of the major players in biofilm formation, it is not the only one. Other microorganisms such as *F. nucleatum, P. gingivalis*, and *S. sanguinis* are also involved in biofilm formation [44]. Thus, more studies on various microorganisms should be conducted to further discuss biofilm.

Multiple firings bring changes in color and translucency due to alterations in crystalline structure and surface specifications [45,46]. The decrease in translucency after firing could be an obstacle in obtaining the desired appearance since additive firing is basically to suffice esthetic purposes by veneering. In this study, the decrease in translucency parameter after firing was between 0.5 and 0.92. According to the previous study, a decrease in translucency parameter as much as 1.24 was translated to an increase of 0.02 in contrast ratio [47]. If the contrast ratio, which is another parameter to determine translucency, is smaller than 0.07, it is known that the difference in translucency is not detectable by human eyes [48]. The decrease in translucency parameter in this study was smaller than 1.24, so the increase in contrast ratio would be even smaller, leading to an undetectable change of translucency. Therefore, despite the decrease in translucency parameter after firing, it was not large enough to be detected. This can be an important advantage since potentially beneficial changes in roughness, contact angle, and *S. mutans* viability, as previously discussed, are achieved not affecting translucency.

The physical changes by multiple firing of zirconia and their potential advantages were discussed in this study. However, it may be necessary to investigate whether multiple firing could affect other factors such as longevity, structural stability, color, and marginal integrity that might influence the function of dental restorations. If there is a negative effect on those factors, the clinical importance of the presented results might be questioned. Thus, additional research that confirms the original function as dental restoration is needed. In addition, the effect of multiple firings without the presence of additives (liner, stain, etc.) might also be investigated to observe possible interfacial interaction. By doing so, it would allow more sophisticated control of the experimental conditions and improve the clinical significance of the current study.

In a dental prosthesis, the veneering material covers most of the outer surface and core material, so two different materials can affect each other. Veneering ceramic increases the bulk thickness and may cause a change in the mechanical strength. According to one study, the cause of failure of the zirconia-based all-ceramic restorations occurred between the two interfaces or inside the veneer ceramic [49]. Thus, the result of firing after applying veneering porcelain may appear different from this study. Also, thermal behavior in the veneered zirconia is also a significant factor since many failures occur due to thermal incompatibility [50,51]. To evaluate thermal behavior in porcelain-zirconia restorations, variations on firing and cooling rate were studied [52]. Rather than constant firing and cooling rate presented in this study, different conditions may be applied to adjust thermal compatibility between zirconia and veneered porcelain. Optimization of the sintering and firing conditions is also important for the other properties discussed in this study. The effect of various sintering and firing conditions on roughness, contact angle, microbial viability, and translucency is still unknown. Though the current conditions are based on the manufacturer’s instructions, further studies may discover better conditions to achieve superior properties.

This study focused on the phenomenon that took place on the surface. However, zirconia goes through phase transformation from tetragonal to monoclinic under stress [53], so structural changes can also be related to surface characteristics and optical properties. Previous studies of zirconia phase transformation were mostly about changes in mechanical properties such as flexural strength [54,55]. Other than phase transformation, but still in the context of structural analysis, the grain size was also known to affect zirconia properties [56]. Therefore, establishing correlation between structural changes and surface characteristics, more specifically in the additive firing process, may add insights to this study.

The reported characteristics of zirconia in the present study, if reinforced by further studies, might be applied to various clinical situations. For example, zirconia implants can go through firings for better hydrophilicity and less *S. mutans* viability to be used with grafting biomaterials to induce bone growth more efficiently [57,58,59] or for immunodeficient patients [60,61,62]. Also, zirconia after firings might have the potential to be used as the tissue engineering scaffold for stem cells in the field of regenerative dentistry [63].

## 5. Conclusions

The additive firing of zirconia after sintering decreased surface roughness, contact angle, *S. mutans* viability, and translucency. The number of firings after the first firing was not found to be critical in surface characteristics, *S. mutans* viability, and optical property. Changes in surface characteristics might have led to decrease in *S. mutans* viability, while the change of translucency was not clinically significant. This study, if reinforced by further research on microbial viability, implies that additive firing may prevent secondary caries under zirconia restorations, not compromising esthetic appearance.

## Figures and Tables

**Table 1:** Surface roughness parameters of the experimental groups (F0–F6).

	Groups						
	F0	F1	F2	F3	F4	F5	F6
**Ra** **(μm)**	0.16 ^A^ (0.15, 0.16)	0.06 ^B^ (0.06, 0.06)	0.04 ^C^ (0.04, 0.04)	0.05 ^CD^ (0.05, 0.05)	0.06 ^BE^ (0.06, 0.06)	0.05 ^BDF^ (0.05, 0.06)	0.05 ^BDG^ (0.05, 0.06)
**Sa** **(μm)**	0.17 ^A^ (0.15, 0.18)	0.07 ^B^ (0.06, 0.07)	0.06 ^C^ (0.06, 0.06)	0.06 ^BCD^ (0.06, 0.06)	0.06 ^BCE^ (0.06, 0.07)	0.06 ^BCF^ (0.06, 0.06)	0.06 ^BCG^ (0.06, 0.07)
**Sz** **(μm)**	2.25 ^A^ (1.95, 2.32)	0.48 ^B^ (0.45, 0.49)	0.45 ^BC^ (0.43, 0.46)	0.47 ^BD^ (0.45, 0.49)	0.48 ^BE^ (0.44, 0.48)	0.55 ^F^ (0.55, 0.57)	0.54 ^BFG^ (0.50, 0.59)
**Sv** **(μm)**	2.09 ^A^ (2.05, 2.40)	0.75 ^B^ (0.59, 0.75)	0.98 ^BC^ (0.63, 1.30)	0.51 ^BD^ (0.43, 0.63)	0.28 ^BE^ (0.28, 0.63)	0.83 ^CEF^ (0.83, 1.02)	0.83 ^BEG^ (0.63, 0.87)

1. Values with the different uppercase superscripts within the rows are significantly different (*p* < 0.05). 2. Interquartile ranges (first quartile, third quartile) are in parentheses.

**Table 2:** Median translucency parameters of the experimental groups (F0–F6).

	Groups						
	F0	F1	F2	F3	F4	F5	F6
**TP**	5.05 ^A^ (5.05, 5.20)	4.39 ^BCDEFG^ (4.34, 4.50)	4.21 ^C^ (4.20, 4,25)	4.43 ^D^ (4.38, 4.44)	4.66 ^E^ (4.58, 4.68)	4.53 ^EF^ (4.51, 4.57)	4.79 ^DEG^ (4.57, 4.80)

1. TP: Translucency parameter. 2. Values with the different uppercase superscripts are significantly different (*p* < 0.05). 3. Interquartile ranges (first quartile, third quartile) are in parentheses.

## Data Availability

Data sharing is not applicable for this article.
